# Cellular Proteostasis During Influenza A Virus Infection—Friend or Foe?

**DOI:** 10.3390/cells8030228

**Published:** 2019-03-09

**Authors:** Mariana Marques, Bruno Ramos, Ana Raquel Soares, Daniela Ribeiro

**Affiliations:** Institute of Biomedicine (iBiMED) and Department of Medical Sciences, University of Aveiro, 3810-193 Aveiro, Portugal; mar.marques@ua.pt (M.M.); brunocramos@ua.pt (B.R.); ana.r.soares@ua.pt (A.R.S.)

**Keywords:** influenza A virus (IAV), virus–host interaction, proteostasis, protein quality control, protein aggregation, unfolded protein response

## Abstract

In order to efficiently replicate, viruses require precise interactions with host components and often hijack the host cellular machinery for their own benefit. Several mechanisms involved in protein synthesis and processing are strongly affected and manipulated by viral infections. A better understanding of the interplay between viruses and their host-cell machinery will likely contribute to the development of novel antiviral strategies. Here, we discuss the current knowledge on the interactions between influenza A virus (IAV), the causative agent for most of the annual respiratory epidemics in humans, and the host cellular proteostasis machinery during infection. We focus on the manipulative capacity of this virus to usurp the cellular protein processing mechanisms and further review the protein quality control mechanisms in the cytosol and in the endoplasmic reticulum that are affected by this virus.

## 1. Introduction

In mammalian cells, protein homeostasis or proteostasis maintenance is assured through an integrated network that ensures efficient biogenesis, folding and assembling of proteins, as well as the degradation of abnormal conformers. Cells are frequently exposed to external stimuli that can disrupt proteostasis leading to the accumulation of misfolded proteins and, under unmitigated chronic stress conditions, to the formation of potentially pathogenic cytotoxic aggregates [1,2]. To counteract the detrimental effect of aberrant protein accumulation, cells have evolved elaborated protein quality control mechanisms that can adapt to the severity of protein damage, repair disturbances in the proteome and re-establish basal homeostasis [3,4]. Distinct surveillance mechanisms that maintain or re-establish proteostasis have been characterized in the cytoplasm, in the endoplasmic reticulum (ER), and in the mitochondria, including protein refolding mechanisms, degradation pathways, and sequestration. The maintenance of cellular proteome homeostasis is crucial to preserve cellular viability and is essential, among other reasons, to ensure healthy aging and to minimize homeostasis distress caused by extrinsic factors [5,6,7]. 

As opportunistic infectious agents, viruses employ several strategies to hijack and control cellular activities, including protein production and processing, in order to efficiently replicate. Multiple viruses specifically alter organelle morphology and dynamics as part of their replication cycle [8,9], as well as lead to the accumulation of misfolded aggregation-prone proteins, which can be toxic to the cell [10,11,12]. Viruses induce the formation of specialized nuclear or cytoplasmic microenvironments, involving an extensive rearrangement of the cellular cytoskeleton and membrane compartments. These virus-induced compartments, generally termed virus factories, are important not only to recruit and concentrate viral and host components and facilitate the molecular interactions required for essential steps of the viral life cycle, but also to control the cellular antiviral defense [13,14,15]. On the other hand, it is often considered that these inclusion bodies may be part of the host antiviral response to infection [11,16]. 

In this review, we summarize and discuss the induced disruption of the cellular machinery, with focus on proteostasis imbalance, throughout the course of influenza A virus (IAV) life-cycle and explore its significance for infection efficiency. Although information on the interplay between influenza B virus (IBV) and host-cell proteostasis is scarce, we have established a parallel, where appropriate, between the two viruses.

## 2. Influenza Virus Genome and Host Translational Machinery

IAV has been the causative agent for most of the annual respiratory epidemics in humans as well as the for the major influenza pandemics in the last century, associated with high morbidity and mortality especially in the elderly [17]. Imunosenescense, combined with the aging-related progressively increased inflammation, is associated with an enhanced susceptibility to severe infections caused by IAV and hinders prevention by vaccination [18]. Knowing that the ability to activate stress responses to preserve proteostasis is gradually compromised with age [7], one can infer a correlation between the higher susceptibility by the elderly to viral infections and the age-related decline on both the antiviral immune responses and the proteostasis maintenance. 

Currently, the permanent risk of influenza epidemics and pandemics is due to the continuous viral antigenic evolution, linked to the accumulation of point mutations within the viral genome or the genetic reassortments of viral genome segments from different viruses or virus strains [19]. For this reason, IAV becomes quickly resistant to virus-directed antiviral treatments; thus, there is a need for an alternative approach that instead targets for virus-exploited host cell factors.

The IAV genome consists of eight single-stranded negative-sense linear RNA segments (ssRNA), encoding for a different number of proteins depending on the virus strain [20]. The eight RNA segments are separately enclosed in the form of ribonucleoprotein complexes (vRNPs), each wrapped in a helical conformation with the viral nucleoprotein (NP) and a trimeric viral RNA-dependent RNA polymerase (RdRp) complex. The virus particles are covered by a lipidic bilayer envelope, spiked with the membrane-spanning viral glycoproteins, hemagglutinin (HA), and neuraminidase (NA). This virus also encodes for structural matrix proteins (M) that link the envelope with the virus core, and non-structural proteins (NS) required for an efficient replication. 

**Figure 1:**
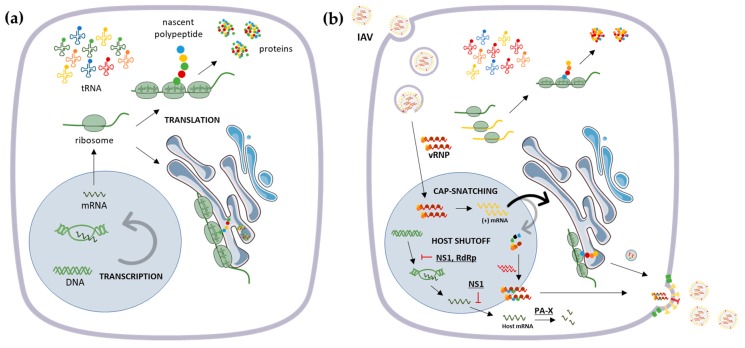
Schematic representation of the transcription and translation machinery in (**a**) uninfected and (**b**) influenza A virus (IAV)-infected cells. (**a**) After being transcribed and processed in the nucleus, mRNA is exported to the cytoplasm where ribosomes initiate their translation. Afterwards, it can either remain free at the cytosol or attach to endoplasmic reticulum (ER) membranes. (**b**) Upon infection, IAV hijacks different stages of mRNA biogenesis and ultimately reduces host protein translation. The virus can also manipulate tRNA pools within the cell to enable the efficient translation of viral proteins. Viral proteins are represented underlined.

To successfully establish infection, viruses can manipulate the host cell to favor the synthesis of viral proteins, at the expense of host proteins, involving changes in translation and RNA synthesis and stability [21,22] (Figure 1). IAV uses various mechanisms of host shutoff to evade the immune response and promote viral replication, even though basal levels of cellular protein synthesis are maintained until the end of the replication cycle to ensure cell survival and, thus, an efficient infection [23].

The viral non-structural protein 1 (NS1) can block the processing of cellular pre-mRNA [24] and targets the polyadenylation tail of nascent cellular transcripts [25,26], which in turn inhibits the nuclear export of mature mRNA and thus their translation. In contrast, the NS1 protein from IBV, as it lacks the carboxyl-terminal effector domain in comparison to IAV NS1, is not capable of inhibiting mRNA polyadenylation or nuclear export [27].

NS1 has also been described to enhance the translation initiation rate of viral mRNA [28], by allowing its preferential translation to the detriment of cellular mRNA [29]. However, contradictory results demonstrate that, although being a general translational enhancer, NS1 does not play a major role in host cell protein synthesis shutoff, suggesting that other IAV products can have a more prominent role in this process [30]. 

NS1 was reported to prevent the activation of the double stranded DNA (dsDNA)-dependent protein kinase R (PKR), both upon IAV and IBV infection [27]. This inhibition is thought to be mediated by the formation of a complex between the two proteins [31,32,33,34]. PKR functions as a gatekeeper of mRNA translation initiation since, upon activation, it phosphorylates the eukaryotic translation initiation factor (eIF2α) decreasing protein synthesis in response to stress signals, mainly as a result of viral infections (reviewed in [35]). However, it is also claimed that PKR activity is not relevant for the preferential translation of viral mRNAs during infection [36]. Besides interfering with PKR, the viral NS1 is known to inhibit host immune responses by limiting the effects of antiviral interferon (IFN) production through the 2′5′-oligoadenylate synthetase (OAS)/RNase L signaling blockage [37]. It is known that RNA cleavage events catalyzed by RNase L are required for optimal inflammasome activation during viral infections [38].

Other mechanisms have been proposed to explain host shutoff by IAV such as cap snatching, in which the virus uses short oligomers from host pre-mRNA to prime its own mRNA synthesis [39]. This process was suggested to be distinct among influenza viruses, since IBV polymerase possesses a distinct cap recognition mechanism in comparison to IAV [40,41]. It has been shown that the IAV viral polymerase complex can target RNA Polymerase II for ubiquitination and degradation, to block host transcription [42]. In addition, viral polymerase acidic protein X (PA-X), expressed from polymerase acidic protein (PA) mRNA as a result of ribosomal frameshifting, degrades target host mRNA both in the nucleus and cytoplasm [43] (Figure 1). It has recently been proposed that IAV-induced shutoff might be a consequence of a reduced abundance of cellular mRNA levels and high levels of viral mRNA within the cell [26], suggesting a viral takeover of the mRNA pool to select the host mRNAs necessary for the virus to efficiently replicate [26,44]. In particular, specific transcripts responsive to stress-induced eIF2α phosphorylation, whose products support adaptive stress, were shown to be translationally induced upon IAV infection [26].

Hence, the dynamic virus–host interactions can elicit alterations in the host proteome to benefit viral replication. Several groups have studied the establishment of temporally and spatially regulated virus–host protein–protein interactions throughout the course of infection, essentially to identify unanticipated cellular factors required for influenza replication and pathogenicity, or related to host antiviral responses, and targetable host factors to guide antiviral drug development [45,46,47,48]. Some of these studies identified abundant proteins in IAV-infected cells as being associated with protein synthesis, chaperone-mediated responses, protein metabolism and modification, including protein folding and proteolysis, and the ubiquitin-proteasome system [45,46,47]. Interestingly, Lietzén et al. (2011) have shown that IAV regulates the expression and/or the subcellular location of host proteins at early phases of infection, suggesting a dynamic interplay between subcellular compartments [48]. Hence, these reports seem to suggest that the characterization of the subcellular proteome, rather than the total proteome, might give more precise insights into whether proteostasis is being disrupted at different stages of the viral life cycle. 

Furthermore, due to the importance of the transfer RNA (tRNA) pool in protein synthesis, viruses have evolved sophisticated strategies to manipulate tRNA populations and optimize tRNA usage to enhance the efficiency of viral translation (reviewed in [49]) (Figure 1). IAV, as well as Vaccinia Virus (VV), were found to manipulate tRNA populations to enable the efficient translation of viral proteins (Figure 1) [50]. The authors demonstrated that, although the total cellular tRNA population remains unchanged upon infection, the polysome-associated tRNA is selectively and significantly altered, suggesting the existence of tRNA pools adapted to viral codon usage upon IAV and VV infections [50]. Interestingly, pandemic strains of IAV, which cause high morbidity and mortality in humans, usually arise as a consequence of the reassortment of avian and human influenza virus’s gene segments, which requires an adaptation of the avian segments to the human hosts. It was recently reported that RNA-directed RNA polymerase catalytic subunit (PB1) protein from the avian influenza pandemic virus H3N2 (1698) evolved in humans to reduce the IFN inhibition by skewing codon usage, partially alleviating the antiviral activities induced by IFN [51]. 

In addition, the IAV has evolved to regulate host cellular microRNAs (miRNAs) to subvert antiviral immunity and escape host-mediated inhibition of viral replication [52,53,54,55]. However, miRNAs can mutually control several processes implicated in IAV replication and pathogenesis by directly binding to its genome (reviewed in [56]). 

## 3. Protein Quality Control Mechanisms and Their Interplay with Influenza A Virus

### 3.1. Cytosolic Responses: Protein Refolding, Degradation and Sequestration

Eukaryotic cells have evolved several cytosolic protein quality control mechanisms that ensure high fidelity on protein synthesis and processing. 

Various chaperones, mainly the heat shock proteins (HSPs), have evolved to assist the efficient folding and assembly of newly synthesized and stress-damaged proteins, in order to prevent potentially pathogenic protein aggregation [57]. The expression of many chaperones is induced upon viral replication, either contributing to the cellular response to infection or facilitating viral propagation. In the latter case, viruses can usurp chaperones by exploiting or modifying their specific roles in order to support infection [10]. Remarkably, some viruses can encode for their own chaperone-like proteins to enhance their infectivity [58].

**Figure 2:**
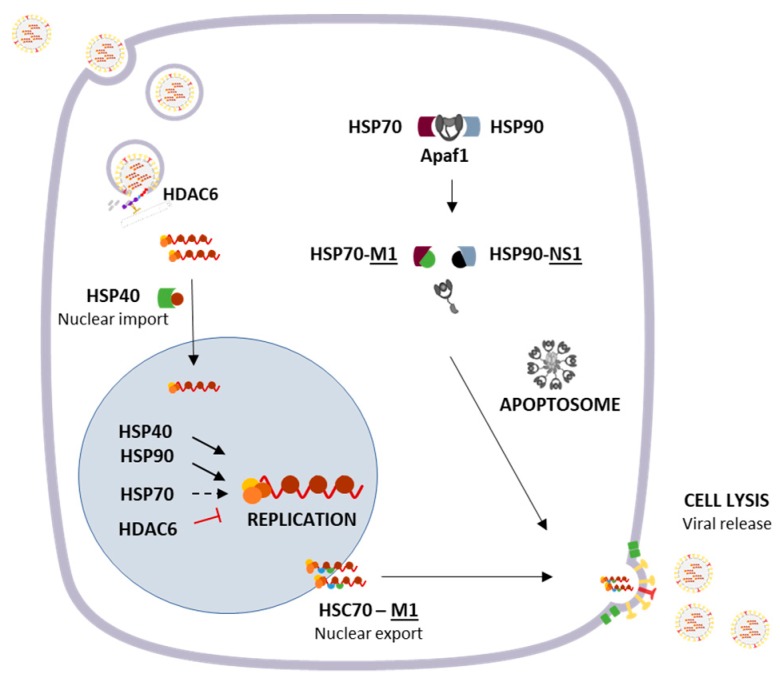
Schematic representation of the interplay between cellular protein chaperones and IAV proteins throughout viral life cycle. Upon attachment with the cellular surface proteins, the virus is internalized by endocytosis and hijacks the cellular aggresome processing machinery (HDAC6) for capsid uncoating and genome release into the cytoplasm. Afterwards, the viral genome (vRNP) is imported to the nucleus together with Hsp40/DnaJB1, where it undergoes transcription and replication. Several host chaperones are involved in mediating viral replication, either by favoring or constraining viral polymerase activity. To spread infection, the virus can additionally induce host cell lysis by inducing apoptosis, a process in which host chaperones are also involved. Dotted lines refer to mechanisms associated to controversial results, as explained in the text. Viral proteins are represented underlined.

The Hsp40/DnaJB1, involved in protein translation, folding, and degradation, has shown to be required for an efficient IAV replication as it interacts at early stages of infection with the viral NP, which encapsidates the viral RNA, and facilitates the nuclear import of incoming viral ribonucleoproteins (RNPs) [59] (Figure 2). 

The Hsp40/DnaJA1 protein can enhance viral RNA synthesis, and ultimately IAV replication, by interacting with the viral RNA polymerase, through its polymerase basic protein 2 (PB2) and PA subunits [60] (Figure 2). Similarly, Hsp70 was previously demonstrated to negatively regulate viral RNP activity [61]. However, a recent study suggests that this protein is crucial for IAV replication and transcription [62]. Both studies support the interaction of Hsp70 with PB1 and PB2, presumably assisting the assembly of the viral polymerase complex and its translocation to the nucleus [61,62]. Nevertheless, Li et al. (2011) demonstrated that high levels of Hsp70 interfere with the integrity of RNP [61], whereas Manzoor et al. (2014) showed that, at normal levels, Hsp70 acts as a chaperone for the viral polymerase [62]. Before, NS1 viral protein was shown to inhibit the processing of Hsp70 pre-mRNA, thus blocking Hsp70 protein expression [63] (Figure 2). Also, Hsp90 was found to relocalize to the nucleus upon IAV infection by interacting with both PB1 and PB2 polymerase subunits. Hsp90 seems to be involved in the assembly and nuclear transport of influenza RNA polymerase subunits [64] and stimulates its RNA synthesis activity [65]. In agreement, Hsp90 inhibitors impairs IAV replication [66], rendering it as a potential target for the treatment of IAV infections (Figure 2). 

At late stages of infection, matrix protein 2 (M2) IAV protein is suggested to form a stable complex with Hsp40 and p58^IPK^, a cellular inhibitor of PKR, consequently enhancing PKR activation [67]. Moreover, the expression of viral NP coincides with the dissociation of p58^IPK^ and Hsp40 to mitigate PKR-mediated antiviral response against IAV infection and ease viral replication [68]. Considering that p58^IPK^ was shown to regulate IAV mRNA translation and promote viral protein synthesis through PKR [69], these reports suggest that the virus can, to some extent, manipulate the regulation of PKR activation depending on the infection stage, though the modulation of Hsp40- p58^IPK^ interaction.

At later times of infection, the heat shock cognate protein 70 (Hsc70), a member of Hsp70 family, was demonstrated to interact with viral matrix protein 1 (M1) in infected cells, showing to be required for the nuclear export of vRNP-M1 complex [70] (Figure 2). 

Interestingly, IAV can modulate apoptosis through the action of both Hsp70 and Hsp90 proteins. In fact, M1 was shown to bind to Hsp70, which results in the disruption of the Hsp70-Apaf-1 complex, consequently leading to apoptotic caspase-9 recruitment, cell lysis, and virus release [71]. Likewise, NS1 protein can interact with Hsp90 to mediate viral-mediated apoptosis through a similar mechanism that involves the weakened interaction between Apaf-1 and Hsp90, thus facilitating the caspase cascade activation [72] (Figure 2). 

Overall, during the course of viral infection, IAV seems to take advantage of several cellular chaperones in different crucial steps of the life cycle, including nuclear import, viral genome replication, nuclear export, and further virion lytic release from the host cell to spread infection. 

**Figure 3:**
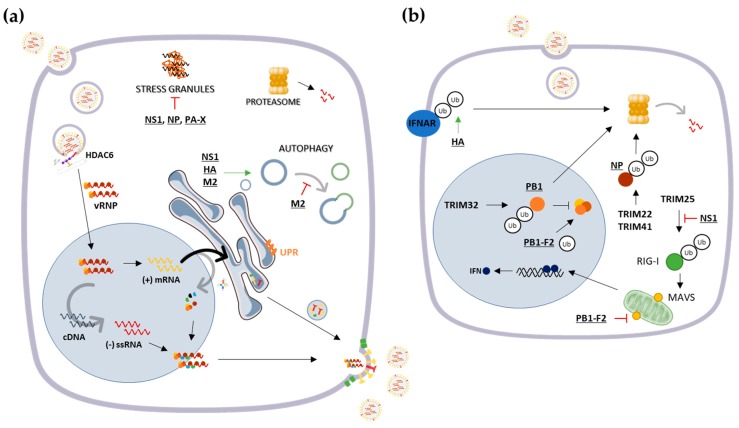
Schematic representation of cellular proteostasis mechanisms and its interplay with IAV proteins during infection. During its life cycle, the virus can interfere with several cellular proteostasis-related mechanisms, namely by (**a**) inhibiting stress granule formation or taking advantage of autophagosome formation, as well as by (**b**) manipulating ubiquitin-proteasome system (UPS)-related proteins to efficiently replicate. Viral proteins are represented underlined.

Alternatively, when proteins cannot be refolded and aggregation cannot be prevented, molecular chaperones may contribute, along with the ubiquitination machinery, to target the misfolded proteins to degradation by the ubiquitin-proteasome system (UPS) or the autophagosome–lysosome pathway (Figure 3). 

A functional UPS is important for the replication of influenza viruses, in order to maintain proper function and the level of viral proteins, although it can act as a host antiviral strategy to mark viral components for degradation [73,74]. Studies have shown that the disruption of proteasome activity using specific inhibitors can lead to the sequestration of incoming influenza particles in cytoplasmic endosomes, directly affecting the early stages of viral replication and blocking the following nuclear import and viral protein expression [75,76]. Widjaja et al. [75] have demonstrated that IAV RNA synthesis depends on UPS and described the ubiquitination process as necessary for IAV entry and replication. 

However, influenza proteins can be targeted for ubiquitin-mediated degradation as an antiviral mechanism employed by the cells. The E3 ubiquitin ligase TRIM32 is known to interact and directly ubiquitinate the PB1 viral protein, leading to its degradation and the subsequent reduction of its polymerase activity [77]. Besides, the TRIM22-mediated polyubiquitination and subsequent proteasome-dependent degradation of NP was also shown to limit IAV replication [78]. More recently, TRIM41 has shown to likewise target viral NP for degradation, to such an extent that TRIM41 deficiency increases host susceptibility to influenza [79] (Figure 3).

On the other hand, IAV has evolved to modulate ubiquitination events to evade immune responses, by degrading or inactivating cellular proteins that limit viral growth and support virus replication. For example, NS1 interferes with the domain of TRIM25 required for RIG-I ubiquitination to circumvent downstream antiviral IFN production [80,81,82], by preventing impeding the formation of RIG-I/MAVS complexes [83] (Figure 3). Another strategy employed by the virus to suppress antiviral IFN responses is by targeting the IFN receptor subunit 1 (IFNAR1) to degradation through its ubiquitination mediated by viral HA [84] (Figure 3).

Yet, the ubiquitination of viral proteins has been shown to be essential for its correct biological activity. Concretely, the ubiquitination of M2 protein has shown to be crucial for the efficient packaging of the viral genome into infectious particles [85]. Similarly, the ubiquitination of IAV NP has been associated with an improvement on viral RNA replication [86,87]. Lastly, posttranslational modifications of PB1-F2 through ubiquitination were revealed to be crucial for the regulation of viral RdRp activity [88] (Figure 3). 

The autophagosome–lysosome pathway is similarly manipulated upon infection with IAV, in a way that the formation of autophagosomes is triggered, but the fusion with lysosomes is inhibited by the virus [40] (Figure 3). Autophagy deficiency was demonstrated to significantly reduce the levels of viral proteins, mRNA, and genomic RNAs without affecting viral entry [89]. In agreement with these observations, autophagy was suggested to be actively involved in IAV replication [90] in a time-dependent manner [91,92,93], since viral production is reduced by the depletion of autophagy-required proteins [90]. In fact, autophagy diminishes the early IFN response to IAV [94] and several viral proteins, namely NS1, HA, and M2, have shown to be involved in the upregulation of autophagy [40,95]. Interestingly, the M2 homologous protein of IBV does not induce autophagosome accumulation [40].

This might imply that the virus benefits from autophagy to accumulate viral elements during replication, without affecting progeny virus production. In fact, it was recently suggested that autophagy plays a role in supporting IAV-induced apoptosis and replication [91]. Interestingly, autophagy deficiency resulted in the impairment of Hsp90 induction in response to IAV infection [89], that has already been referred as crucial for an efficient replication cycle. Considering these results, it seems that IAV takes advantage of both proteasome and the autophagosome systems to efficiently replicate, in particular to allow the nuclear import of influenza particles and to permit its genome replication. 

When the amount of misfolded proteins in cells exceeds the refolding or degradative capacity of the quality-control machinery, or when this machinery is disrupted, the misfolded or aggregated proteins may be sequestered and compartmentalized into stress foci to minimize their toxic effects and interference with the normal cellular functions [96]. Upon viral infection, the formation of specialized sites for viral replication can also result in specific imbalances in protein folding control and the formation of insoluble protein aggregates. 

In response to immune activation, rather than impaired proteolysis, the formation of aggresome-like induced structures (ALIS) in the cytosol is also induced as an early event in the cellular adjustment to proteostasis disruption, consisting in the temporary aggregation of ubiquitinated proteins [97]. In IAV-infected bone marrow-derived dendritic cells, the induced delay in antigen presentation coincides with the accumulation of newly synthesized ubiquitinated proteins in large and transient cytosolic structures [98] (Figure 3). 

On the other hand, the IAV PB1-F2 protein was shown to form highly cytotoxic amyloid-like fibers in IAV-infected cells. The accumulation of these fibers can be a crucial virulence factor and presumably one of the causes for the immunopathological disorders observed during IAV infections [99]. During the replicative cycle, the PB1-F2 protein can also target the mitochondrial inner membrane space and consequently impair the RLR-dependent antiviral signaling by binding to MAVS and inhibiting IFN expression [100,101] (Figure 3). 

Thus, IAV seems to induce the formation of inclusion bodies to escape the host cellular antiviral response and induce pathogenicity. In specific cases, the presence of RNA sensors, viral RNA, and MAVS in infected cells within cytoplasmic inclusion bodies correlates with the inhibition of an antiviral IFN-mediated response [102]. A mechanism of viral evasion of innate immunity via spatial isolation of TBK1/IKKε into inclusion bodies was suggested in cells infected with another negative-sense ssRNA virus [103,104], thus suppressing the IRF3-mediated RLR antiviral signaling and IFN induction [103]. However, it is still not known whether IAV can also induce this accumulation of key players from the host’s innate immunity to evade the antiviral response.

Upon viral infection, the deregulation of proteostasis may lead to the accumulation of insoluble misfolded proteins that, along with chaperones and proteasome components, are often targeted to juxtanuclear structures termed aggresomes. These proteins are actively transported in a retrograde dynein-based manner along the microtubule cytoskeleton to the perinuclear site at the microtubule-organizing center, where they are sequestered into aggresomes [105]. It involves the microtubule-associated histone deacetylase 6 (HDAC6), which can promote the autophagic clearance of protein aggregates and protect cells from cytotoxic accumulation of misfolded proteins, acting as an adaptor that binds polyubiquitin chains from substrates and dynein [106] (Figure 2). 

Whether IAV leads to the accumulation of misfolded proteins is still not known. Yet, it is known that IAV usurps the HDAC6 ubiquitin-binding function, taking advantage of the aggresome processing machinery for capsid disassembly and uncoating during entry in cell [107]. The fusion pore formed between the viral envelope and the endosomal membrane exposes the viral core that contains unanchored ubiquitin chains to the cytosol, which might activate HDAC6 similarly to aggresome processing, culminating with the viral genome release into the cytoplasm to be further imported to the nucleus. Recently, it was found that HDAC6 restrict IAV transcription and replication by deacetylation of the viral RNA polymerase PA subunit, promoting its proteasomal degradation [108], suggesting that HDAC6 has a dual role in sustaining and constraining IAV replication.

Alternatively, misfolded proteins strongly tend to accumulate in stress granules (SGs), stress-induced cytosolic ribonucleoprotein complexes that assemblies when translation is inhibited. By sequestering polyribosome-associated components, SGs can reprioritize translation and rout the stalled components for re-initiation or degradation. The continuing accumulation of misfolded proteins can affect SGs dynamics, promoting its conversion into an aberrant aggresome-like structure that can be eventually transported to aggresomes and further degraded. The presence of cellular chaperones, specifically HSP27 and HSP70, has shown to prevent the formation of aberrant SGs and promote SGs disassembly by helping to maintain its normal composition and dynamism [109] (Figure 3).

It was already reported that the formation of SGs was not observed upon infection with IAV, suggesting that this virus can efficiently prevent global translation arrest [110]. However, in cells infected with the same viral strain comprising specific mutations on the NS1 protein, SGs start to accumulate at a time post infection that coincides with a strong activation of PKR, a well-known translation inhibitor [110]. Since NS1 is previously shown to prevent PKR activation [31], the inhibition of SG formation might be in part dependent on the function of NS1. 

Besides NS1, NP and PA-X contribute to block the SG assembly [111,112]. In fact, IAV NP was shown to be crucial for the P58^IPK^ activation and subsequent inhibition of PKR-mediated host response during IAV infection by exploiting Hsp40 [68]. Remarkably, the formation of SGs in virus-infected cells inversely correlates with accumulation of viral gene products, indicating that SG formation might be an important part of the cellular antiviral response. 

### 3.2. The Endoplasmic Reticulum and the Unfolded Protein Response

In eukaryotic cells, the ER is the major site for protein folding and trafficking. Therefore, ER proteostasis perturbations, including those caused by viral infection, may lead to accumulation of misfolded proteins and their aggregation within the organellar lumen, which induces stress in the ER [113]. Its ability to facilitate protein folding renders ER chaperones as essential and the major facilitators of proper virus assemblage. For IAV, the folding and maturation efficiency of viral glycoproteins in the ER, namely HA and NA, impact the production of viral particles [114]. Concretely, the binding of ER calnexin and calreticulin facilitates the IAV HA productive folding [115]. 

Once a threshold of misfolded protein accumulation in the ER has been reached, it activates the unfolded protein response (UPR) (reviewed in [116]). As an immediate response, general protein translation is attenuated through the activation of PKR-like ER kinase (PERK) to decrease the load of newly synthesized proteins into ER. Simultaneously, the activation of inositol-requiring enzyme 1 (IRE1) leads to the preferential degradation of mRNA encoding for ER-localized proteins via IRE1-dependent decay (RIDD), and to an unconventional splicing of the transcription factor X box-binding protein 1 (XBP1) mRNA that controls the expression of UPR-related genes. Lastly, the activation of activating transcription factor 6 (ATF6) pathway also contributes to the synthesis of stress-attenuating proteins, as chaperones and folding catalysts. In addition, the ER-associated protein degradation (ERAD) may also be activated, which allows the clearance of misfolded and accumulated proteins in the ER, and, if these steps fail, the UPR might ultimately lead to apoptosis. 

Upon infection, the UPR might either be mobilized by the host in an attempt to restrict viral propagation or be manipulated by the virus to benefit its replication. For instance, the innate sensing of viruses by ER stress was found to be responsible for the pathogenicity of pandemic IAV [117]. On the other hand, the ER-sensing of viral glycoproteins, namely the viral HA protein, which mediates the attachment of the viral particle to the host cell and escape from the endosome, triggers an antiviral immunity response that inhibits IAV replication, resulting in the rapid proteasomal-degradation of this protein mediated by the ERAD pathway [118]. 

**Figure 4:**
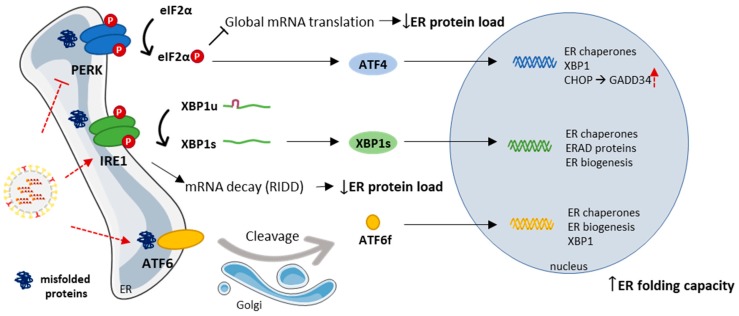
Schematic representation of the three main branches of unfolded protein response (UPR) in the ER (PERK, IRE1, and ATF6). In normal conditions, the activation of UPR helps to diminish ER stress by reducing ER protein load and increasing ER folding capacity. To evade the antiviral effects of PKR, the viral NS1 has been shown to inhibit protein kinase R (PKR) activity, which in turn will affect the PERK-induced response. Previous reports, although contradictory, also demonstrate that upon infection the virus can to some extent influence the mediated signaling of the three UPR branches. Red dotted arrows represent still controversial actions of IAV on UPR targets. Viral proteins are represented underlined.

The role of the UPR during IAV infection is unclear, since the results on the subject are somewhat controversial. Hassan et al. [119] reported that IAV induces ER stress responses through IRE1, with little or no concomitant activation of the PERK and the ATF6 pathway, and further showed that IAV causes a significant induction of several ER-stress response-related genes, including CHOP and GADD34. Inversely, Roberson et al. [120] demonstrated that IAV can induce ER-stress via ATF6 activation, but not CHOP. Yet, chemical genomics identified PERK as a cellular target for influenza virus inhibition, as, according to the authors [121], IAV can lead to the attenuation of PERK-mediated UPR, but does not distress neither ATF6 or IRE1 arms, in contrast to Hassan and Roberson’s results [119,120]. It is important to consider though that the approaches used by the groups are distinct and further analysis are needed in order to fully understand the response mediated by the UPR in the course of IAV infection (Figure 4). 

## 4. Concluding Remarks

Upon IAV infection, several processes within the cytosolic and the ER protein quality control mechanisms, namely protein folding, degradation and sequestration, have shown to contribute either as part of an effective replication cycle or as part of an effective cellular antiviral response to the virus. IAV seems to take advantage of the protein quality control components to replicate and spread infection, although some of the observed effects may also be part of the establishment of cellular reactions to counteract the virus. Some reports show contradictory findings and further studies are needed in order to obtain more clarity and insight into the control of cellular proteostasis mechanisms by both the virus and the host cell.
